# Cooperative Synthesis of Ultra Long-Chain Fatty Acid and Ceramide during Keratinocyte Differentiation

**DOI:** 10.1371/journal.pone.0067317

**Published:** 2013-06-27

**Authors:** Yukiko Mizutani, Hui Sun, Yusuke Ohno, Takayuki Sassa, Takeshi Wakashima, Mari Obara, Kohei Yuyama, Akio Kihara, Yasuyuki Igarashi

**Affiliations:** 1 Laboratory of Biomembrane and Biofunctional Chemistry, Faculty of Advanced Life Science, Hokkaido University, Sapporo, Japan; 2 Laboratory of Biochemistry, Faculty of Pharmaceutical Sciences, Hokkaido University, Sapporo, Japan; University of Geneva, Switzerland

## Abstract

The lipid lamellae in the stratum corneum is important for the epidermal permeability barrier. The lipid lamellae component ceramide (CER), comprising an ultra long-chain (ULC) fatty acid (FA) of ≥26 carbons (ULC CER), plays an essential role in barrier formation. ULC acyl-CoAs, produced by the FA elongase ELOVL4, are converted to ULC CERs by the CER synthase CERS3. In the presented study, we observed that *ELOVL4* and *CERS3* mRNAs increased during keratinocyte differentiation *in vivo* and *in vitro*. We also determined that peroxisome proliferator-activated receptor β/δ is involved in the up-regulation of the mRNAs. Knockdown of *CERS3* caused a reduction in the elongase activities toward ULC acyl-CoAs, suggesting that CERS3 positively regulates ULCFA. Thus, we reveal that the two key players in ULC CER production in epidermis, CERS3 and ELOVL4, are coordinately regulated at both the transcriptional and enzymatic levels.

## Introduction

The outermost layers of the epidermis are responsible for critical protective functions, including permeability barrier functions against water loss [1], [2]. The epidermal permeability barrier is localized in the extracellular domains of the stratum corneum (SC), where a hydrophobic lipid mixture is organized into distinct multi-lamellar membrane structures composed primarily of free fatty acids (FAs), cholesterol, and ceramides (CERs) [3], [4]. The balance of these lipids in SC is, in general, important for skin functions, and their production should be commonly regulated. However, any mechanism behind such regulation still remains unclear.

The sphingolipid backbone CER consists of a long-chain base attached to a FA via an amide bond, which is catalyzed by the enzymatic actions of a CER synthase. Each CER synthase exhibits a distinct tissue distribution pattern and FA chain length specificity [5], [6]. The FA’s carbon chain length defines its classification, with long-chain (LC) FAs having a carbon chain length of 11–20 (C11–C20), very long-chain (VLC) FAs, more than 20 (>C20), and ultra long-chain (ULC) FAs more than 24 (>C24). In mammalian tissues, the most common CERs are LC CERs with C16∶0 FA and VLC CERs with C24 (C24∶0 or C24∶1) FAs [7], [8]. The greatest molecular heterogeneity in mammalian CERs is observed in the epidermis, which expresses at least 11 species [3], [5], [9]. One unique CER in the epidermis is ω-hydroxy-CER, which carries an ULCFA and covalently binds to linoleic acid to form ω-*O*-acylCER, or to cornified envelope proteins such as involucrin [3], [5], [10], [11]. In epidermis, newly synthesized CERs are immediately converted to glucosylCERs and sphingomyelins, which are then packaged into lamellar bodies in the stratum granulosum (SG). Both can be converted back to their CER species, via hydrolysis occurring at a site lying between the SG and SC, then CER is incorporated into the lipid lamellae in the SC [3].

In *de novo* synthesis of CER, dihydrosphingosine is *N*-acylated by CER synthases to produce dihydroCER [7]. To date, six mammalian CER synthases (CERS1-6) have been identified and characterized [5], [6]. These CERS family members differ in their tissue-specific expression patterns and their preferences toward acyl-CoAs [5], [6]. For example, CERS5 and CERS6 produce LC CERs, mainly C16 [12]. CERS2 and CERS3 demonstrate a preference for VLCFA, although expression of CERS2 is ubiquitous, whereas CERS3 expression is tissue specific and occurs mainly in skin and testis [5], [13], [14]. In addition, CERS3 is up-regulated during human keratinocyte differentiation, as we previously reported [15]. CERS3 also exhibits activity toward several acyl-CoAs including VLC acyl-CoA [13]. Therefore, we speculated that CERS3 would be important in the synthesis of VLC CERs/ULC CERs in the human epidermis.

VLCFAs are synthesized from LCFAs by FA elongation, which occurs by cycling through a 4 step process (condensation, reduction, dehydration, and reduction) [8]. The FA elongase machinery comprises four distinct enzymes including the FA elongases, which catalyze the first, rate-limiting step. To date, seven elongases (ELOVL1–7) have been identified and characterized in mammals [8], [16], [17]. Each elongase exhibits a characteristic substrate specificity toward FA chain length and degree of saturation. For example, ELOVL1 is responsible for the production of C24∶0 and C24∶1 acyl-CoAs, which are primarily used for C24 CER/sphingolipid production [17]. ELOVL4 plays roles in the generation of ULC acyl-CoAs, the precursors of the ULC CERs that function in skin barrier formation [18], [19], [20], [21], [22].

Peroxisome proliferator-activated receptors (PPARs) are members of the nuclear receptor family of transcription factors. The three PPAR isoforms that exist in mammals, PPARα, PPARβ/δ, and PPARγ, regulate the expression of numerous genes involved in lipid metabolism [23]. PPARβ/δ is the most abundant of the PPAR isoforms in fetal epidermis [24], and it has a known role in SC formation and permeability barrier development [25]. In addition, levels of epidermal barrier-related CERs like ω-*O*-acylCER are increased by the activation of PPARβ/δ, at least in the hairless mouse [26]. Conversely, PPARβ/δ knockout mice exhibit a significant delay in barrier recovery after either acetone treatment or tape-stripping [27].

In the presented study, we investigated the regulation of the production of ULCFA and ULC CERs, and the cooperation between the two during human keratinocyte differentiation. We examined ELOVL family members during keratinocyte differentiation and analyzed changes in their ULCFA-synthesizing activity and mRNA expression. We also examined the regulation of ULCFA synthesis exerted by ULC CER synthesis. Our results suggest that the production of VLCFAs/ULCFAs in differentiated keratinocytes is regulated by CERS3. Furthermore, we demonstrated that the activation of PPARβ/δ stimulates CERS3 and ELOVL4 expression in human keratinocytes.

## Materials and Methods

### Cell Culture and Transfection

Normal human epidermal keratinocytes isolated from neonatal skin were obtained from Cambrex (Walkersville, MD) and grown in a serum-free keratinocyte growth medium (Invitrogen, Carlsbad, CA) containing 0.07 mM calcium. Keratinocyte differentiation was performed as described previously using a differentiation medium, a mixture of DMEM medium (Sigma, St Louis, MO) and Ham F-12 medium (2∶1, v/v), supplemented with 1.3 mM calcium, 10% FBS, 10 µg/ml insulin, 0.4 µg/ml hydrocortisone, and 50 µg/ml vitamin C [15].

Human embryonic kidney (HEK) 293T cells were grown in DMEM medium containing 10% FBS, in a humidified atmosphere of 5% CO_2_ at 37°C. Transfections were performed using LipofectAMINE Plus Reagent (Invitrogen), according to the manufacturer's directions.

Keratinocytes were treated with vehicle control (DMSO) or a selective transcriptional activator: for RAR, all-*trans*-retinoic acid (Sigma); RXR, 9-*cis*-retinoic acid (Sigma); PPARα, WY14643 (Sigma); PPARβ/δ, L-165,041 (Sigma); PPARγ, troglitazone (Cayman Chemical, Ann Arbor, MI); LXR, TO901317 (Cayman Chemical); and 1α, 25-dihydroxyvitamin D3 (Biomol, Plymouth Meeting, PA).

### Real-time Quantitative PCR

Total RNA was isolated from cultured keratinocyte using a FastPure RNA kit (Takara Bio, Shiga, Japan), then converted to cDNA using a PrimeScript RT reagent kit (Takara Bio) according to the manufacturer’s protocol. Real-time quantitative PCR was performed using a SYBR Premix EX Taq kit (Takara Bio) on an Mx3000 Real Time PCR System (Agilent Technologies, La Jolla, CA). The 10 µl PCR reaction mixture included 1 µl (10–20 ng) cDNA, 5 µl SYBR Premix Ex Taq, 0.2 µl 50x ROX Reference DyeII, and 0.2 µl each of the forward and reverse primers (10 µM). The nucleotide sequences of the primers used for *ELOVL4* were 5′-TTTGGTGGAAACGATACCTGA-3′ and 5′-AGTGCATCCATTTGGGGAAG-3′; for *ELOVL5*, 5′-GCACATTCCCTCTTGGTTGG-3′ and 5′-GGAGGCCCCTTTCTTGTTGT-3′; for *ELOVL6,* 5′-GCTAAGCAAAGCACCCGAAC-3′ and 5′-GGTGATACCAGTGCAGGAAGA-3′; for *keratin 5* were 5′-CTCAGTGGAGAAGGAGTTGGAC-3′ and 5′-CTGCCACTGCCATATCCAGA-3′; and for *keratin 10,* 5′-TGAGACGTAATGTACAAGCTCTGG-3′ and 5′-CGGTTTCAGCTCGAATCTGT-3′. Other primers were purchased from Takara Bio (*ELOVL1*, HA140347; *ELOVL7*, HA138981; *CERS2*, HA109841; *CERS3*, HA063716; *PPARβ/δ*, HA140209; and *GUSB*, HA067813). The transcript level of each gene was normalized with that of the housekeeping gene *GUSB*, encoding β-glucuronidase. Each reaction mixture was incubated at 95°C for 30 sec, followed by 40 cycles at 95°C for 5 sec and 60°C for 30 sec.

### RNA Interference

Commercially available siRNAs for human *CERS3* (Hs-LASS3-5) and human *PPARβ/δ,* (Hs-PPARβ-3, -6, and -11), and control siRNA were all purchased from Qiagen (Cambridge, MA). Keratinocytes were transfected with the appropriate siRNA (5–20 nM) using HiPerFect transfection reagent (Qiagen) according to the manufacturer’s instruction. Knockdown of the target gene was confirmed by real-time quantitative PCR.

### *In vitro* FA Elongation Assays

*In vitro* FA elongation assays were performed as described previously [17]. The extracted lipids were separated either by normal-phase TLC after conversion of acyl-CoA products to FAs or by reverse-phase TLC after conversion of acyl-CoAs to FA methyl esters. Labeled lipids were detected and quantified using an FLA7000 bioimaging analyzer (Fuji Photo Film, Tokyo, Japan) or BAS-2500 bioimaging analyzer (Fuji Photo Film).

### *In situ* Hybridization

To construct RNA probes for *in situ* hybridization, *keratin 14*, *involucrin*, *Elovl4*, and *CerS3* cDNAs were amplified by PCR using primers: for *keratin 14*, primers K14-1 (5′-TGAACCGCGAGGTGGCCACCAACAG-3′) and K14-2 (5′-TTAGTTCTTGGTGCGCAGGACCTGC-3′); for *involucrin*, primers Inv-1 (5′-CCCTGTGAAGGATCTGCCTG-3′) and Inv-2 (5′-GGTTCCTGACACTCCTGGTG-3′); for *Elovl4*, primers E4-1 (5′-GAGGAAGAAAAACAACCAAGTCTCC-3′) and E4-2 (5′-AATTTACTCTCCTTTTGGCTTCCCG-3′); and for *CerS3*, primers C3-1 (5′-TGGTTCTGGTCGGAGAGATACTGGC-3′) and C3-2 (5′-GAAGCTCATCAGACTAATAGCAGCC-3′). The amplified cDNA fragments were cloned into the pGEM-T Easy vector (Promega, Madison, WI), and each anti-sense RNA was transcribed from the SP6 promoter and were labeled with digoxygenin using a DIG RNA labeling mix (Roche Applied Science, Indianapolis, IN) and SP6 RNA polymerase (Roche Applied Science).

*In situ* hybridization was performed as essentially described elsewhere [28]. Briefly, skin samples isolated from E18.5 mouse embryos were fixed with 4% paraformaldehyde and hybridized with the digoxygenin-labeled RNA probe. After washes, the hybridized probe was detected using an alkaline phosphatase-conjugated anti-digoxygenin antibody (F_ab_ fragment; Roche Applied Science) and subsequent signal development for 6–24 h in a nitroblue tetrazolium/5-bromo-4-chloro-3-indolyl phosphate solution. The sample was post-fixed overnight with 3.7% formaldehyde in sodium phosphate buffer (pH 7.4), equilibrated in 30% sucrose, and frozen in Tissue-Tek OCT compound (Sakura Finetek, Alphen aan den Rijn, Netherlands). Sections were cut into 25 µm sections using a cryostat (CM3050S, Leica Biosystems, Wetzlar, Germany) then were covered with a glass coverslip using CC/Mount (Diagnostic Biosystems, Pleasanton, CA). Images were captured using a DM5000B light microscope (Leica Biosystems) equipped with a DFC295 digital color camera (Leica Biosystems).

### Immunoblotting

Total cell lysates prepared from keratinocytes were subjected to SDS-PAGE, transferred to PVDF membranes, and then reacted with anti-CERS3, anti-CERS2 (M02A, Abnova, Taiwan), anti-involucrin (sc-28557, Santa Cruz Biotechnology, Santa Cruz, CA), anti-actin (A2066, SIGMA) or anti-GAPDH (FL-335, Santa Cruz Biotechnology). Anti human CERS3 polyclonal rabbit antibodies were generated in rabbits using a synthetic multi-antigen peptide corresponding to 18 amino acids of human CERS3 (88-KHSTRQPLQTDIYGLAKK-105). Human CERS3 polyclonal antibody was purified by immunoaffinity column chromatography. A 1∶5000 dilution of horseradish peroxidase-conjugated donkey anti-mouse or rabbit IgG F(ab')_2_ fragment (GE Healthcare Life Sciences, Piscataway, NJ) was used as the secondary antibody. Labeling was detected using ECL Western Blotting Detection Kit (GE Healthcare Life Sciences).

## Results

### ELOVL4 is Up-regulated during Keratinocyte Differentiation

Mammalian epidermis contains unique CERs, including ULC CERs. We previously demonstrated that *CERS3* mRNA is highly expressed in keratinocytes and is up-regulated during keratinocyte differentiation [15]. Therefore, we speculated that CERS3 plays important functions in the synthesis of VLC CERs/ULC CERs, and in the regulation of VLCFA/ULCFA production, in differentiated keratinocytes.

**Figure 1:**
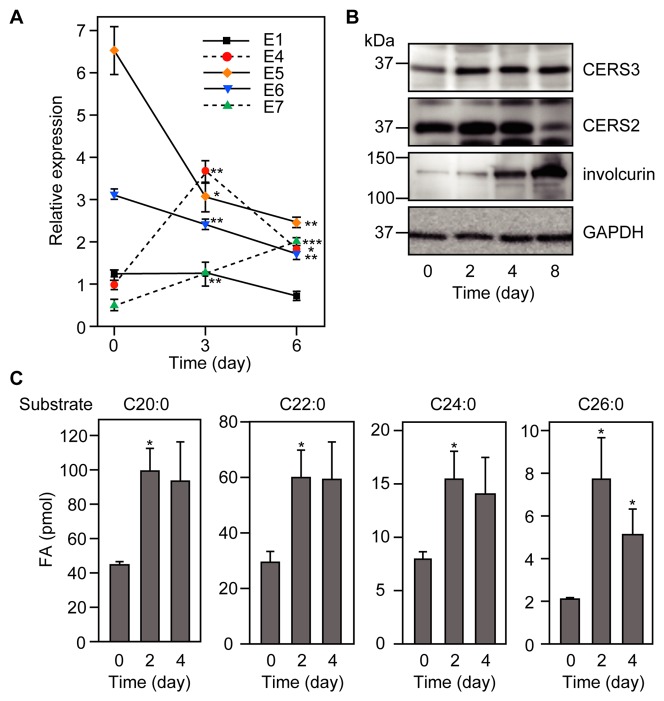
ELOVL4 is up-regulated during keratinocyte differentiation. (A) Total RNA was prepared from keratinocytes differentiated for 0, 3, or 6 days in differentiation medium. SYBR green-based real-time quantitative PCR was performed using primers specific for *ELOVL1*, *ELOVL4*, *ELOVL5, ELOVL6*, or *ELOVL7*, and for *GUSB* as an internal control. The expression level of each *ELOVL* mRNA was calculated using a standard curve and normalized to that of *GUSB*. Values presented are the amount of each *ELVOL* mRNA relative to that of *ELOVL4* at day 0, and represent the mean ± S.D. from three independent reactions. Statistically significant differences from day 0 are indicated (*p<0.05, **p<0.01, ***p<0.001; Student’s t-test). E1, *ELOVL1*; E4, *ELOVL4*; E5, *ELOVL5*; E6, *ELOVL6*; E7, *ELOVL7*. (B) Total cell lysates (10 µg protein) prepared from keratinocytes differentiated for 0, 2, 4, or 8 days in differentiation medium were subjected to immunoblotting with an anti-CERS3 antibody, anti-CERS2 antibody, anti-involucrin antibody, or, to demonstrate uniform protein loading, anti-GAPDH antibody. (C) Total membrane proteins (40 µg protein) from keratinocytes differentiated for 0, 2, or 4 days were incubated with the indicated acyl-CoA (50 µM) and 0.075 µCi [^14^C] malonyl-CoA for 30 min at 37°C. After termination of the reactions, acyl-CoAs were converted to FAs and separated by normal-phase TLC, followed by detection and quantification by a BAS-2500 bioimaging analyzer (Fuji Photo Film). Values presented are FA levels and represent the mean ± S.D. from three independent experiments. Statistically significant differences compared to 0 day cells are indicated (*p<0.05; Student’s t-test).

Of the seven ELOVL family members (ELOVL1-7), human primary keratinocytes express five: *ELOVL1, ELOVL4, ELOVL5, ELOVL6,* and *ELOVL7*. We examined changes in the mRNA expression levels for these five during keratinocyte differentiation, using real-time quantitative RT-PCR (Fig. 1A). ELOVL5 is a polyunsaturated FA-specific elongase; the other four ELOVLs have been implicated in the production of saturated ULCFAs [8], [16], [17]. ELOVL6 elongates C16 to C18, ELOVL7 mainly elongates C18 to C20, ELOVL1 generates C20-C28, and ELOVL4 produces ≥C26 FAs [17]. The expression of *ELOVL4* mRNA increased from day 0 to day 3 after transfer to differentiation medium (Fig. 1A; see also Fig. S1B). Although the *ELOVL4* mRNA gradually decreased, the expression of *ELOVL4* mRNA still remained higher compared to that before differentiation even at day 6 (Fig. 1A). The expression of ELOVL4 in epidermis and its importance for barrier function has been reported using a mouse model [18], [19], [20], [21]. The stratum basale (SB) marker keratin 5 was down-regulated from day 0 to day 4, while the stratum spinosum (SS) marker keratin 10 was increased from day 2 to day 4 (Fig. S1E and F). The SG marker involucrin also was increased from day 4 to day 8 (Fig. 1B). These results suggest that *ELOVL4* mRNA expression is high in SS and SG. The expression levels of *ELOVL1* mRNA remained nearly unchanged during the differentiation (Fig. 1A and Fig. S1A). *ELOVL7* mRNA was slightly increased through day 6, whereas *ELOVL5* and *ELOVL6* mRNAs were decreased from day 0 to day 6 (Fig. 1A). The prominent up-regulation of the *ELOVL4* mRNA may be responsible for the production of substantial levels of ULCFAs required for cutaneous barrier functions.

Next we performed *in vitro* FA elongase assays, using total membrane fractions prepared from undifferentiated (day 0) and differentiated keratinocytes (day 2 and day 4) and C24∶0-CoA, and C26∶0-CoA as substrates, to detect ULCFA elongation activity (Fig. 1C). Consistent with the expression profile of the *ELOVL4* mRNA, the elongase activities toward C24∶0-CoA and C26∶0-CoA were increased from day 0 to day 4. Separation of the products by reverse-phase TLC indicated that the primary products from the substrates C24∶0-CoA and C26∶0-CoA were C26∶0-CoA and C28∶0-CoA, respectively (Fig. S2). The elongase activities toward C20∶0-CoA and C22∶0-CoA were also increased during differentiation (Fig. 1C), although the expression levels of *ELOVL1* mRNA were nearly unchanged during differentiation (Fig. 1A and Fig. S1A).

We previously reported that *CERS3* mRNA is up-regulated during the course of keratinocyte differentiation [15] (see also Fig. S1D). Here, we confirmed the increase in CERS3 protein levels by immunoblotting (Fig. 1B). In contrast to CERS3 protein levels, the mRNA and protein levels of CERS2, which is responsible for VLC CERs production, decreased from day 4 to day 8 (Fig. 1B and Fig. S1C).

**Figure 2:**
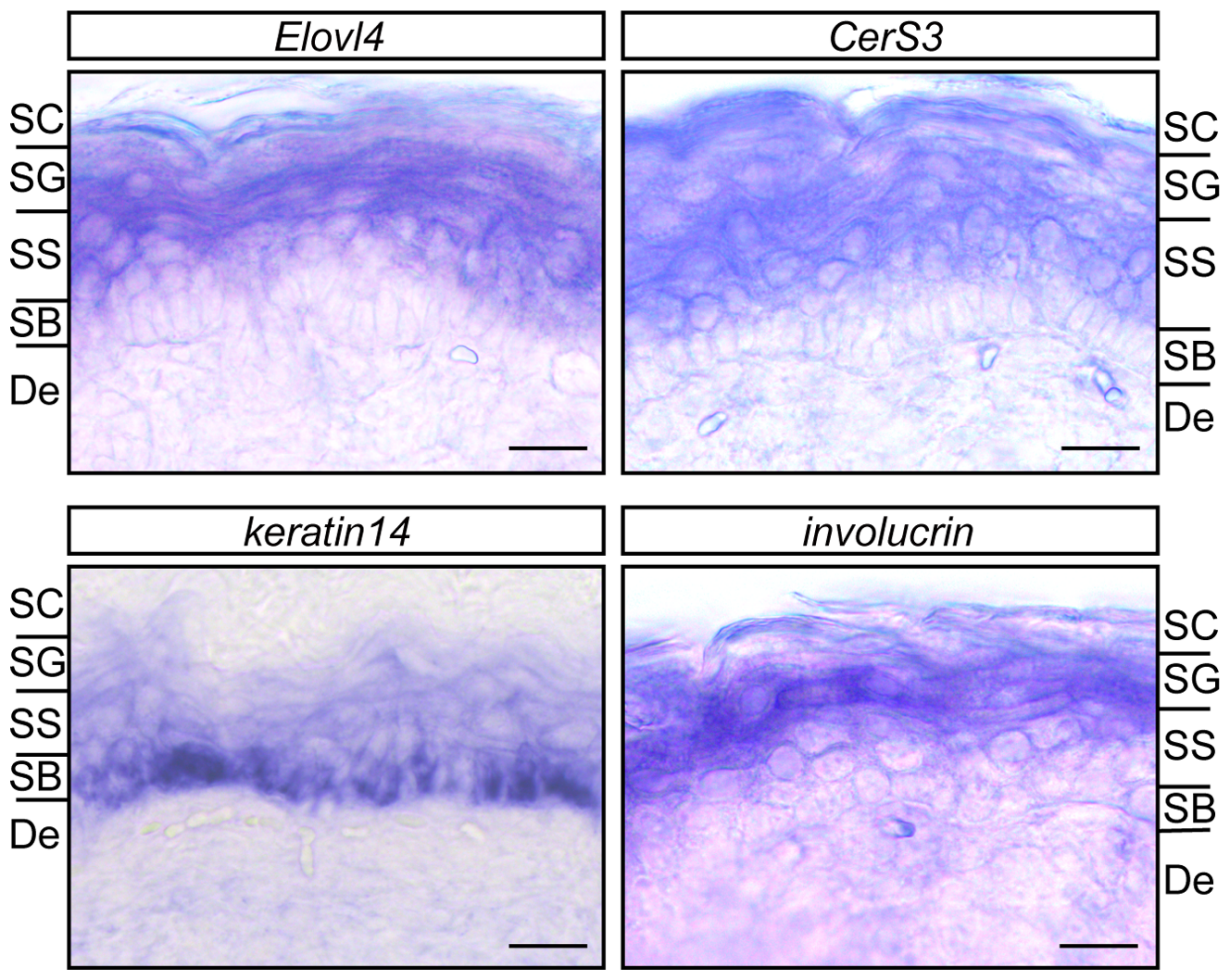
Expression of *Elovl4 and CerS3* mRNAs in the SS and SG. Skin samples isolated from E18.5 mouse embryos were fixed with 4% paraformaldehyde, hybridized with a digoxygenin-labeled RNA probe for *Elovl4*, *CerS3*, *keratin 14*, or *involucrin*, and stained with alkaline phosphatase-conjugated anti-digoxygenin antibody (F_ab_ fragment) and nitroblue tetrazolium/5-bromo-4-chloro-3-indolyl phosphate solution. Frozen sections (25 µm) were subjected to microscopic observation under a DM5000B light microscope and photographed. Bar, 20 µm. De, dermis.

Next, we examined the up-regulation of *Elovl4* and *CerS3* mRNAs *in vivo* using mouse skin. *In situ* hybridization assays revealed that both *Elovl4* and *CerS3* mRNAs were expressed in SS and SG but were absent in SB (Fig. 2). The SB and SG layers were confirmed by staining of their specific markers *keratin 14* and *involucrin*. Expression of *CerS3* mRNA in SS and SG is consistent with previous study [29]. In summary, *ELOVL4* and *CERS3* levels increase during keratinocyte differentiation, and these increases may be responsible for the production of ULC CERs/sphingolipids in the epidermis.

### CERS3 Regulates VLCFA and ULCFA Synthesis in Keratinocytes

**Figure 3:**
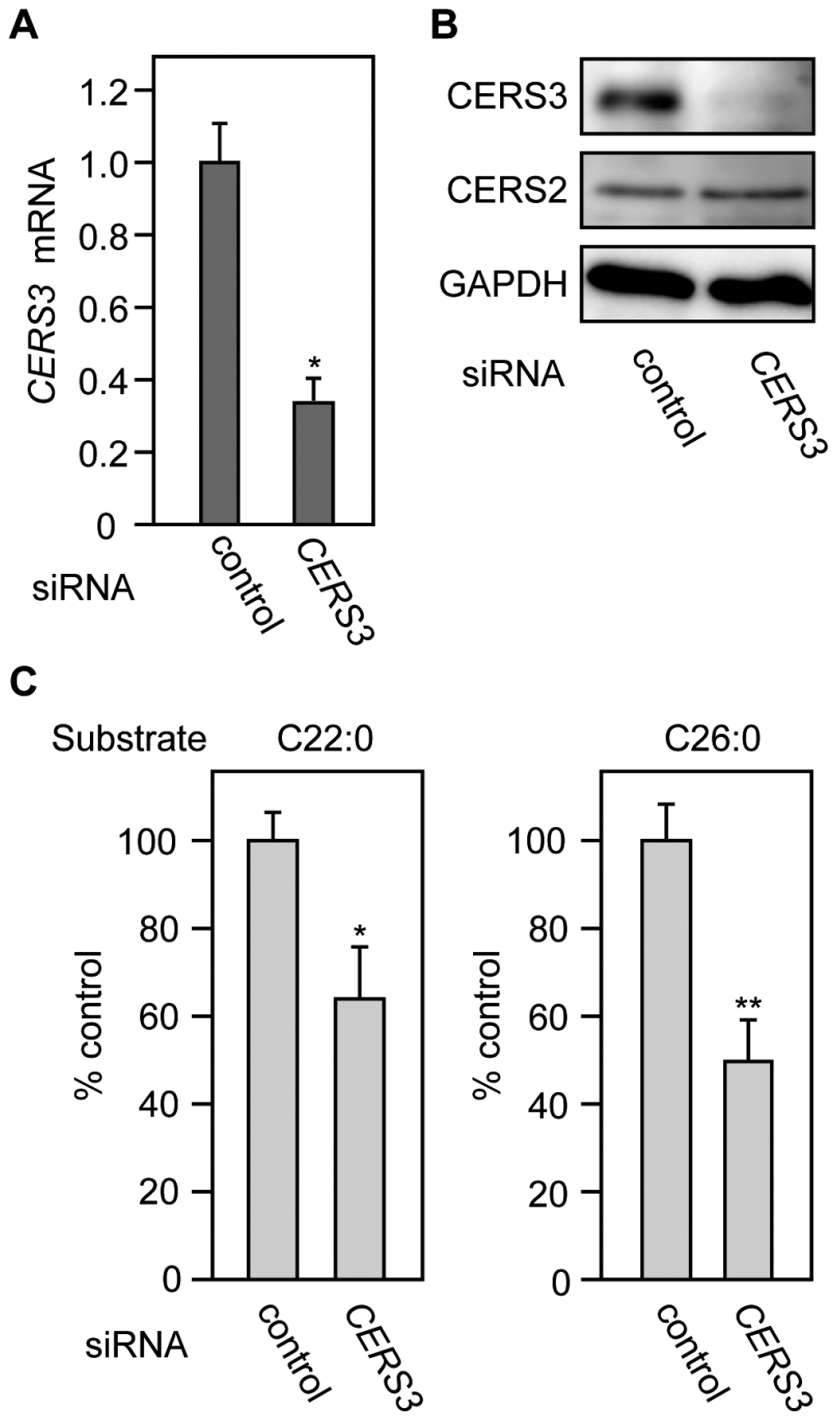
CERS3 regulates activities of ELOVL1 and ELOVL4 in differentiated keratinocyte. (A–C) Keratinocytes were transfected with control or *CERS3* siRNA. Twenty four h after transfection, transfection media were changed to differentiation medium, and the cells were incubated for an additional 2 days. (A) Total RNA was prepared from the cells, then subjected to real-time PCR using primers specific for *CERS3* and *GUSB* as an internal control. The expression level of *CERS3* mRNA was calculated by normalizing to that of *GUSB*. Values presented are the amount of *CERS3* mRNA relative to that in control cells and represent the mean ± S.D. from three independent experiments. Statistically significant differences to control siRNA are indicated (*p<0.05; the Student’s t-test). (B) Total cell lysates (10 µg protein) prepared from the cells were subjected to immunoblotting with an anti-CERS3 antibody or anti-CERS2 antibody, or with an anti-GAPDH antibody to demonstrate uniform protein loading. (C) Total membrane proteins (40 µg) were prepared and subjected to an *in vitro* FA elongase assay by incubating with 50 µM C22∶0-CoA or C26∶0-CoA and 0.075 µCi [^14^C]malonyl-CoA, for 30 min at 37°C. After termination of the reactions, lipids were subjected to methanolysis, extraction, separation by reverse-phase TLC, and detection by an FLA7000 bioimaging analyzer. Values indicate the radioactivities of the FA methyl ester products relative to that of control siRNA-transfected cells, and represent the mean ± S.D. of three independent experiments. Statistically significant differences to control siRNA are indicated (*p<0.05, **p<0.01; Student’s t-test).

In lysates from differentiated keratinocytes, we observed increases in elongase activities toward C20∶0-CoA and C22∶0-CoA, for which ELOVL1 is responsible (Fig. 1C). However, the mRNA expression levels for *ELOVL1* were not greatly increased, but in fact appeared nearly unchanged during the differentiation (Fig. 1A and Fig. S1A). We previously revealed positive regulation of ELOVL1 by CERS2 using HeLa cells [17]. Such regulation may lead to cooperation between the production of VLC CERs and the production of VLCFAs. In keratinocytes, CERS3 is upregulated during differentiation, which might imply that CERS3 plays important roles in the production and regulation of both VLC CERs and ULC CERs in keratinocyte differentiation. To examine the potential roles of CERS3, we performed knockdown of *CERS3* during keratinocyte differentiation, using siRNAs. *CERS3* mRNA prepared from differentiated keratinocytes (day 2) was decreased to ∼35% in cells treated with *CERS3* siRNA treatment, compared to controls (Fig. 3A). *CERS3* siRNA caused a reduction in the protein levels of CERS3, but not in those of CERS2 (Fig. 3B). Furthermore, *CERS3* siRNA treatment decreased FA elongation activities toward both C22∶0-CoA and C26∶0-CoA (Fig. 3C). These results suggest that CERS3 regulates ELOVL1 and ELOVL4 activities in keratinocytes.

### PPARβ/δ is Involved in the Up-regulation of CERS3 and ELOVL4 during Keratinocyte Differentiation

**Figure 4:**
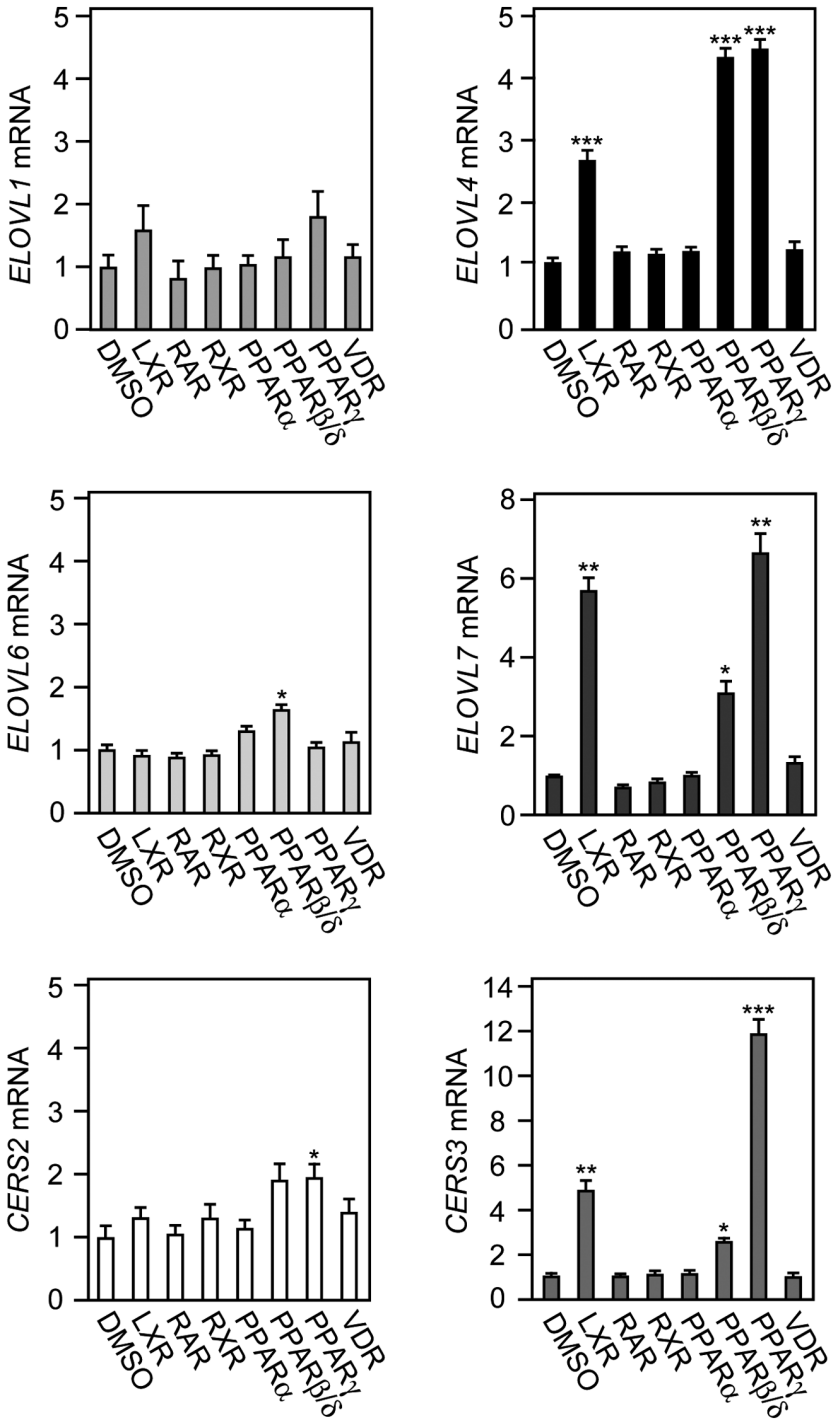
Activation of PPARβ/δ, PPARγ, or LXR increases *CERS3, ELOVL4,* and *ELOVL7* mRNA expression in keratinocytes. Keratinocytes were incubated for 24 h with vehicle (DMSO) or with an activator of the indicated transcription factor: LXR (10 µM TO901317), RAR (1 µM all-*trans*-retinoic acid), RXR (1 µM 9-*cis*-retinoic acid), PPARα (10 µM WY14643), PPARβ/δ (10 µM L-165,041), PPARγ (7.5 µM troglitazone), or vitamin D receptor (VDR) (0.1 µM 1α, 25-dihydroxyvitamin D3). Total RNA prepared from each culture was subjected to real-time quantitative PCR using primers specific for *ELOVL1*, *ELOVL4*, *ELOVL6*, *ELOVL7*, *CERS2*, or *CERS3* and for *GUSB* for standardization. The expression level of each mRNA was calculated by normalizing to that of *GUSB*. Values presented are the amount of the respective mRNA relative to that from cells treated with vehicle, and represent the mean ± S.D. from three independent experiments. Statistically significant differences to DMSO controls are indicated (*p<0.05; **p<0.01; ***p<0.001; Student’s t-test).

To determine the transcription factors responsible for the up-regulation of CERS3 and ELOVL4 during keratinocyte differentiation, we examined the effects of activators specific for several nuclear receptor family members, including LXR, PPARα, PPARβ/δ, PPARγ, RXR, RAR, and vitamin D receptor. Treatment of keratinocytes with an activator for PPARβ/δ, PPARγ, or LXR strongly induced the expression of *ELOVL4* mRNA (Fig. 4). The same set of activators was also effective for the induction of *ELOVL7* and *CERS3* mRNAs. In contrast, the mRNA expression levels of *ELOVL1*, *ELOVL6*, and *CERS2*, each of which were not induced during keratinocyte differentiation, were mostly unchanged following treatment with either of the nuclear receptor family activators. However, slight increases were observed in *ELOVL6* mRNA following PPARβ/δ activation, and in the *CERS2* mRNA following PPARγ activation (Fig. 4). The other activators tested had no effect on any of the mRNA expression levels.

**Figure 5:**
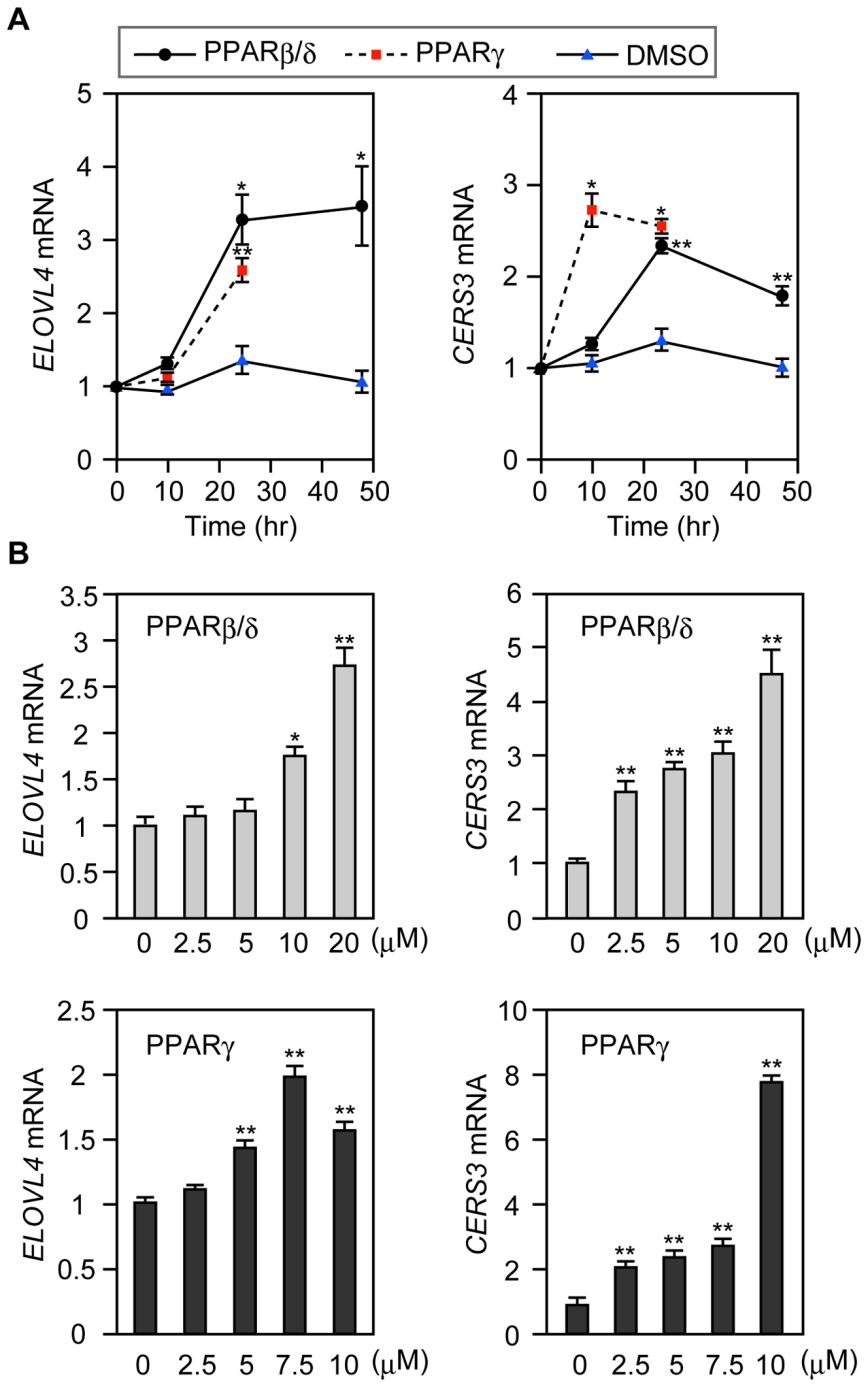
Treatment with ligands for PPARβ/δ or PPARγ increase *CERS3* and *ELOVL4* mRNA expression in keratinocytes, in both a dose- and time-dependent manner. (A) Keratinocytes were incubated with either DMSO or activators of the indicated receptor, PPARβ/δ (10 µM L-165,041) or PPARγ (7.5 µM troglitazone), for the indicated times. (B) Keratinocytes were incubated for 24 h with the indicated concentrations of the PPARβ/δ activator L-165,041 or the PPARγ activator troglitazone. (A and B) Total RNA prepared from each culture was subjected to real-time PCR using primers specific for *ELOVL4* or *CERS3*, and for *GUSB* for standardization. The expression level of each mRNA was calculated by normalizing to that of *GUSB*. Values presented are the amount of the respective mRNA relative to that from cells harvested at 0 h (A) or from cells not treated with activator (B), and represent the mean ± S.D. from three independent experiments. Statistically significant differences to the samples at 0 h (A) or untreated samples (B) are indicated (*p<0.05, **p<0.01; Student’s t-test).

We examined the effects of the PPARβ/δ and PPARγ activators on *ELOVL4* and *CERS3* mRNA expression in more detail. The up-regulation of *ELOVL4* mRNA by PPARβ/δ and PPARγ ligands occurred rapidly and was saturated after 24 h (Fig. 5A). The effects of these ligands were dose-dependent, although a slight decrease was observed with the PPARγ activator at 10 µM (Fig. 5B). The induction of *CERS3* mRNA expression by the PPARγ activator occurred more rapidly, compared to that observed for the *ELOVL4* mRNA expression, and was dose-dependent (Fig. 5A and B). The time course of the *CERS3* mRNA expression following treatment with the PPARβ/δ ligand was similar to that for the *ELOVL4* mRNA. For both, the expression levels of the *CERS3* mRNA reached maximum, 24 h after treatment. However, the effective concentration of the PPARβ/δ ligand was lower for *CERS3* mRNA expression than for *ELOVL4* mRNA expression. We also confirmed that the CERS3 protein levels were increased by PPARβ/δ, PPARγ, and LXR activators (Fig. S3A and B). In summary, activating PPARβ/δ, PPARγ, or LXR stimulates *CERS3* and *ELOVL4* mRNA expression. These results suggest that these three transcription factors have important roles in VLC CER and ULC CER synthesis in keratinocytes.

### PPARβ/δ is Involved in the Induction of CERS3 and ELOVL4 mRNA Expression during Keratinocyte Differentiation

**Figure 6:**
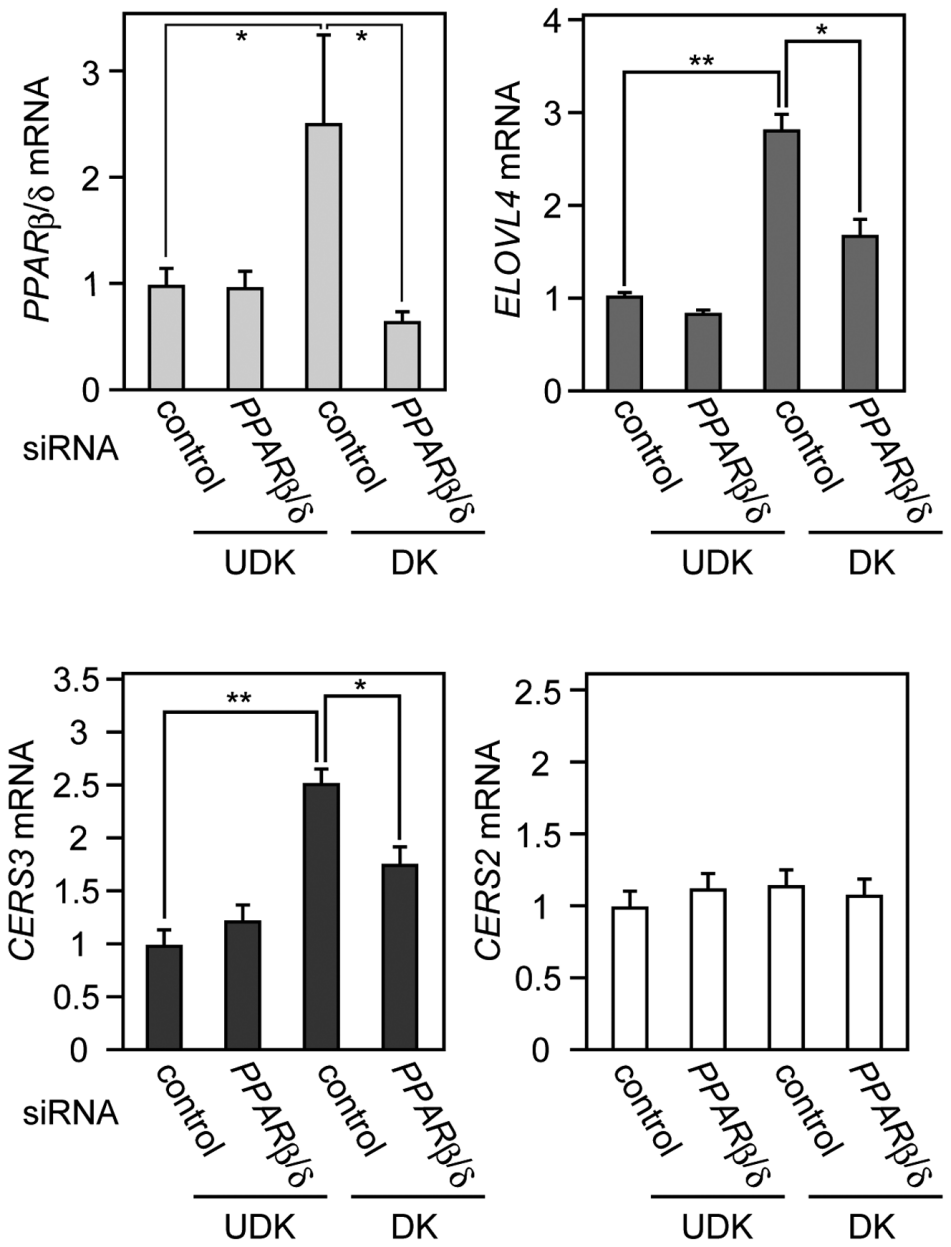
Knockdown of *PPARβ/δ* by siRNA attenuates differentiation-induced *ELOVL4* and *CERS3* mRNA expression in keratinocytes. Keratinocytes were transfected with control or *PPARβ/δ* siRNA. Twenty four h after transfection, the transfection media were changed to serum-free keratinocyte growth medium (undifferentiated keratinocytes, UDK) or to differentiation medium (differentiated keratinocytes, DK), and cells were incubated for another 2 days. Total RNA prepared from each of the cell cultures was subjected to real-time quantitative PCR using primers specific for *PPARβ/δ*, *ELOVL4*, *CERS3*, or *CERS2*, and for *GUSB* for standardization. The expression level of each mRNA was calculated by normalizing to that of *GUSB*. Values presented are the amount of the respective mRNA relative to that from undifferentiated keratinocytes transfected with control siRNA, and represent the mean ± S.D. from three independent experiments. Statistically significant differences between undifferentiated keratinocytes and differentiated keratinocytes or between control siRNA and *PPARβ/δ* siRNAs are indicated (*p<0.05, **p<0.01; Student’s t-test).

*PPARβ/δ* knockout mice exhibit a significant delay in recovery after skin barrier disruption [27]. In addition, the activation of PPARβ/δ stimulates both CER synthesis and lamellar body formation in keratinocytes [26]. With these points in mind, we next focused on the involvement of PPARβ/δ in the mRNA expression of *CERS3* and *ELOVL4*. We first examined whether *PPARβ/δ* expression in keratinocytes was affected by differentiation. We also investigated whether *PPARβ/δ* knockdown by siRNA could attenuate the increases in *CERS3* or *ELOVL4* mRNA expression observed in the early stage of keratinocyte differentiation (day 2). *PPARβ/δ* mRNA expression was increased during differentiation by ∼2.5 fold (Fig. 6). Furthermore, treatment with siRNA efficiently caused a 74% reduction in the induced *PPARβ/δ* mRNA levels. Even more remarkable, increases in *CERS3* and *ELOVL4* mRNA expression observed during differentiation were both significantly inhibited by the *PPARβ/δ* siRNA, whereas the *CERS2* mRNA levels were not affected (Fig. 6). We also observed a reduction in CERS3 protein levels by the *PPARβ/δ* siRNA (Fig. S3C). These results indicate that the increases in *CERS3* and *ELOVL4* mRNA levels during keratinocyte differentiation are mediated, at least in part by *PPARβ/δ.*

## Discussion

As the outermost layer of the epidermis, the SC is responsible for permeability barrier function [1], [2]. The extracellular lipid lamellae in SC, comprising free FAs, CERs, and cholesterol, provide the structure of the permeability barrier [3], [4]. Among the lamellar lipids, CERs, especially ULC CERs, are particularly important for barrier function [3], [5], and the FA elongase ELOVL4 and the CER synthase CERS3 play central roles in ULC CER production [18], [19], [20], [21], [29]. Considering the apparent cooperation in the production of ULC CERs, we had speculated that the synthesis of ULCFAs and the synthesis of ULC CERs might be tightly regulated. However, such regulation has been largely undetermined. In the presented study, we found that the mRNA expression levels of *ELOVL4* and *CERS3* are up-regulated during keratinocyte differentiation both *in vitro* and *in vivo* (Figs. 1 and 2 and Fig. S1), and that PPARβ/δ is involved in this up-regulation (Figs. 4, 5 and 6). Our results also suggest that the activity of ELOVL4 is regulated by CERS3 (Fig. 3). Thus, ULCFA production and ULC CER production are concertedly regulated at both the transcriptional and enzymatic levels in keratinocytes.

We have demonstrated that in skin, *CERS3* mRNA is the most abundant of the CERS members [15]. Furthermore, its expression is up-regulated during keratinocyte differentiation. In addition, CERS3 reportedly exhibits enzyme activities toward several VLC and ULC acyl-CoAs, including C26∶0-CoA [13], [29]. Considering this information, we propose that CERS3 is responsible for ULC CER production in epidermis. Indeed, it was recently reported that *CerS3* knockout mice are completely deficient in the production of ULC CERs (≥C26), and that these mice exhibit severe skin barrier defects [29]. Interestingly, this phenotype resembles that of *Elovl4* knockout mice. *Elovl4* knockout mice are also defective in the production of ULCFAs (≥C28) and the unique ω-*O*-acylCER in skin, and these defects lead to neonatal death due to transepidermal water loss [18], [19], [20], [21]. A pathological importance of *CERS3* and *ELOVL4* was also recently reported. Recessive mutations in these genes cause ichthyosis characterized by impaired skin barrier functions [30], [31].

During keratinocyte differentiation, the production of C24 VLCFAs, and C26 and C28 ULCFAs increases (Fig. 1C). C24 VLCFA production is catalyzed by ELOVL1 [17]. However, *ELOVL1* mRNA levels were nearly unchanged during keratinocyte differentiation (Fig. 1A and Fig. S1A). Therefore, we speculate that ELOVL1 activity, but not its expression level, increases during differentiation. We previously reported that ELOVL1 activity is positively regulated by the VLC CER synthase CERS2 [17]. When HeLa cells were treated with *CERS2* siRNA, ELOVL1 activity was decreased. Although CERS2 is responsible for almost all VLC CER production in most tissues, this is not so in epidermis. The VLC CER composition of epidermis in *CerS2* knockout mice is similar to that in wild type mice, and *CerS2* knockout mice exhibit no unusual skin phenotype [29]. In addition to CERS2, then, CERS3 may also catalyze VLC CER synthesis in epidermis and compensate the production of VLC CER in the *CerS2* knockout mice. Our previous *in vitro* analysis indicated that CERS3 is active toward C24∶0-CoA [13]. We found that CERS3 is important for the regulation of VLCFA production (Fig. 3). Knockdown of *CERS3* using siRNA caused a reduction in C24 VLCFA production. Thus, in keratinocytes CERS3 plays central roles both in VLC CER production and in the regulation of the production of the VLCFA used in VLC CER synthesis. We demonstrated here that CERS3 also regulates ULCFA production (Fig. 3).

We speculate that regulation of VLCFA or ULCFA production may be mediated by an interaction of CERS3 with ELOVL1 or ELOVL4, respectively, similar to the regulation of VLCFA synthesis by CERS2, which we recently reported [17]. In general, verification for interactions among membrane proteins, especially when the proteins are overproduced, is difficult, so future studies will be required to address this point. In our current model, CERS3 may facilitate the release of the acyl-CoA products initialized by ELOVL1 or ELOVL4, thereby preventing the products from being stuck within the elongase complex; unreleased acyl-CoA would inhibit the next round of the elongation cycle. However, we cannot exclude another possibility, that changes in lipid composition due to altered CERS3 levels lead to the observed ELOVL1 or ELOVL4 activities.

We further observed a concerted expression of *CERS3* and *ELOVL4* mRNAs during keratinocyte differentiation (Figs. 1 and 2 and Fig. S1), which may be important for the production of the abundant ULC CERs required for skin barrier formation. This concerted expression could be achieved by using a common transcription factor. In the presented study, we further demonstrated that activators for PPARβ/δ, PPARγ, and LXR induced the expression of both *CERS3* and *ELOVL4* mRNAs (Fig. 4). Several lines of evidence indicate that PPARβ/δ plays an important role in regulating gene expression, differentiation, lipid accumulation, and wound healing in epidermis [27], [32], [33]. Furthermore, treatment of cultured keratinocytes with activators of PPARβ/δ is known to increase the mRNA and protein levels of markers of keratinocyte differentiation, such as involucrin [33]. We demonstrated here *PPARβ/δ* siRNA inhibited the induction of *CERS3* and *ELOVL4* mRNAs during differentiation (Fig. 6). These results suggest that PPARβ/δ is a key transcription factor for the concerted expression of *CERS3* and *ELOVL4* mRNAs during keratinocyte differentiation.

We also detected the induction of *ELOVL4, ELOVL7*, and *CERS3* mRNA expression in keratinocytes by treatment with an LXRα ligand (Fig. 4). Recently, several reports revealed cross-talk between LXR and PPARs in regulating FA metabolism [34]. It was also reported that an LXR ligand activated the expression of all three subtypes of PPAR and their downstream target genes in the human keratinocyte cell line HaCaT [35], suggesting that PPARs mediate the lipogenic function of LXR. We also detected the induction of PPARs by the LXRα ligand in primary human keratinocytes (data not shown).

In summary, we revealed here that the production of ULC CER and that of its constituent ULCFA are concertedly regulated in the epidermis. Skin permeability barrier function is maintained by the extracellular lipid lamellae and cornified envelope. Future studies are needed to determine how the components of extracellular lipid lamellae, as well as cornified envelope proteins, are coordinately regulated to orchestrate such well-organized structures.

## Supporting Information

Figure S1 **The expression levels of ELOVL4 and CERS3 are up-regulated during keratinocyte differentiation.** (A–F) Total RNA prepared from keratinocytes differentiated for 0, 2, 4, or 6 days in differentiation medium was subjected to real-time quantitative PCR using primers specific for *ELOVL1* (A), *ELOVL4* (B), *CERS2* (C), *CERS3* (D), *keratin 5* (*K5*; E), or *keratin 10* (*K10*; F), and for *GUSB* for standardization. The expression level of each mRNA was calculated by normalizing to that of *GUSB*. Values presented are the amount of the respective mRNA relative to that from cells harvested at day 0, and represent the mean ± S.D. from three independent experiments. Statistically significant differences to the value at day 0 are indicated (*p<0.05, **p<0.01, ***p<0.001; Student’s t-test).
(TIF)

Figure S2 **Determination of the chain-lengths of FAs produced by** ***in vitro*** **FA elongation assays using keratinocytes.** Total membrane proteins (40 µg) prepared from keratinocytes differentiated for the indicated days were incubated with C24∶0-CoA or C26∶0-CoA (50 µM) and 0.075 µCi [^14^C] malonyl-CoA for 30 min at 37°C. After termination of the reactions, lipids were subjected to methanolysis, extraction, separation by reverse-phase TLC, and detection using an FLA7000 bioimaging analyzer (Fuji Photo Film).
(TIF)

Figure S3 **CERS3 protein expression in keratinocytes is regulated by PPARβ/δ.** (A) Keratinocytes were incubated with either DMSO, PPARγ activator (7.5 µM troglitazone), or LXR activator (10 µM TO901317) for 24 h. Total cell lysates (5 µg protein) were subjected to immunoblotting with an anti-CERS3 antibody, or, to demonstrate uniform protein loading, an anti-actin antibody. (B) Keratinocytes were incubated with either DMSO or PPARβ/δ activator (10 µM L-165,041) for 24 h. Total cell lysates (2 µg protein) were subjected to immunoblotting with an anti-CERS3 antibody or anti-actin antibody. (C) Keratinocytes were transfected with control or *PPARβ/δ* siRNA. Twenty four h after transfection, medium was changed to differentiation medium. Cells were incubated for another 2 days. Total cell lysates (10 µg protein) were prepared and subjected to immunoblotting with an anti-CERS3 or anti-actin antibody.
(TIF)

Information S1 (DOC)
