# Supplementary material for: Cooperative Synthesis of Ultra Long-Chain Fatty Acid and Ceramide during Keratinocyte Differentiation
Source: PLoS One. 2013 Jun 27;8(6):e67317. doi: 10.1371/journal.pone.0067317 (PMC3694974; doi:10.1371/journal.pone.0067317)
Supplement: Information S1 — (DOC) [file pone.0067317.s004.doc]

**Supporting Information**

**Supplemental Experimental Procedures**

**Primer sets for real-time quantitative PCR**

The nucleotide sequences of the primers used for *keratin 5* were 5’-CTCAGTGGAGAAGGAGTTGGAC-3’ and 5’-CTGCCACTGCCATATCCAGA-3’; and for *keratin 10,* 5’-TGAGACGTAATGTACAAGCTCTGG-3’ and 5’-CGGTTTCAGCTCGAATCTGT-3’.

**Supplemental Figure Legends**

**Figure S1. The expression levels of ELOVL4 and CERS3 are up-regulated during keratinocyte differentiation.** (A-F) Total RNA prepared from keratinocytes differentiated for 0, 2, 4, or 6 days in differentiation medium was subjected to real-time quantitative PCR using primers specific for *ELOVL1* (A), *ELOVL4* (B), *CERS2* (C)*, CERS3* (D)*, keratin 5* (*K5*; E), or *keratin 10* (*K10*; F), and for *GUSB* for standardization. The expression level of each mRNA was calculated by normalizing to that of *GUSB*. Values presented are the amount of the respective mRNA relative to that from cells harvested at day 0, and represent the mean ± S.D. from three independent experiments. Statistically significant differences to the value at day 0 are indicated (*p < 0.05, **p < 0.01, ***p<0.001; Student’s t-test).

**Figure S2. Determination of the chain-lengths of FAs produced by *in vitro* FA elongation assays using keratinocytes.** Total membrane proteins (40 μg) prepared from keratinocytes differentiated for the indicated days were incubated with C24:0-CoA or C26:0-CoA (50 μM) and 0.075 μCi [^14^C] malonyl-CoA for 30 min at 37 ˚C. After termination of the reactions, lipids were subjected to methanolysis, extraction, separation by reverse-phase TLC, and detection using an FLA7000 bioimaging analyzer (Fuji Photo Film).

**Figure S3. CERS3 protein expression in keratinocytes is regulated by PPARβ/δ.** (A) Keratinocytes were incubated with either DMSO, PPARγ activator (7.5 μM troglitazone), or LXR activator (10 μM TO901317) for 24 h. Total cell lysates (5 μg protein) were subjected to immunoblotting with an anti-CERS3 antibody, or, to demonstrate uniform protein loading, an anti-actin antibody. (B) Keratinocytes were incubated with either DMSO or PPARβ/δ activator (10 μM L-165,041) for 24 h. Total cell lysates (2 μg protein) were subjected to immunoblotting with an anti-CERS3 antibody or anti-actin antibody. (C) Keratinocytes were transfected with control or *PPARβ/δ* siRNA. Twenty four h after transfection, medium was changed to differentiation medium. Cells were incubated for another 2 days. Total cell lysates (10 μg protein) were prepared and subjected to immunoblotting with an anti-CERS3 or anti-actin antibody.
